# Deep Generative and Graph-Based Representation Learning for Multiomics Survival Stratification in Ovarian Cancer: Secondary Analysis

**DOI:** 10.2196/89069

**Published:** 2026-09-21

**Authors:** Carlos Marino, Claudia Diaz Paz

**Affiliations:** 1 Pontificia Universidad Católica del Perú, Lima, Peru; 2 CENTRUM Católica Graduate Business School, Jirón Daniel Alomía Robles 125, Lima, 15023, Peru, 51 626 7100

**Keywords:** ovarian cancer, personalized medicine, multiomics, variational autoencoders, graph convolutional neural networks, survival analysis

## Abstract

**Background:**

Ovarian cancer remains one of the most lethal gynecologic malignancies, largely due to pronounced molecular heterogeneity, nonspecific clinical presentation, and frequent diagnosis at advanced stages. Multiomics profiling—including genomics, transcriptomics, and epigenomics—offers a powerful avenue for characterizing this complexity and enabling more precise patient stratification.

**Objective:**

This study aimed to address key challenges in multiomics analysis, including high dimensionality, cross-modality heterogeneity, limited sample size, and the lack of effective approaches for survival stratification of patients with ovarian cancer through deep representation learning.

**Methods:**

We analyzed multiomics data from The Cancer Genome Atlas and developed a 5-stage deep learning pipeline centered on variational autoencoders (VAEs) for nonlinear dimensionality reduction and latent representation learning. A graph convolutional neural network component is described as a proposed extension for modeling interaction-aware representations but was not empirically evaluated in this study. Latent embeddings derived from the VAE were clustered using k-means, and their prognostic relevance was assessed using Cox proportional hazards modeling and Kaplan-Meier survival analysis.

**Results:**

Following correction of a clinical-molecular harmonization issue, the final matched cohort comprised 291 patients. Silhouette analysis identified k=2 as the optimal clustering solution (silhouette=0.272). Kaplan-Meier analysis demonstrated significantly different overall survival between the two clusters (log-rank *χ*^2^_1_=10.0; *P*=.002). Cox proportional hazards modeling estimated a hazard ratio of 0.519 (95% CI 0.343‐0.785; *P*=.002), indicating that patients assigned to cluster 1 exhibited an approximately 48% lower hazard of death than those in cluster 0. These results demonstrate that the learned latent representations capture prognostically relevant structure within the integrated multiomics data.

**Conclusions:**

The proposed VAE-based framework identified 2 prognostically distinct patient subgroups with significantly different overall survival. These findings demonstrate the potential of deep generative representation learning for multiomics-based survival stratification in ovarian cancer and provide a foundation for future validation in independent cohorts and the evaluation of graph-based extensions.

## Introduction

### Background

Ovarian cancer ranks eighth among cancers affecting women worldwide in both incidence and mortality [1,2], with an estimated 324,603 new cases and 206,956 deaths in 2022 [1,2]. Although other cancers affecting women, including breast and cervical cancer, have a higher global incidence, ovarian cancer remains a major cause of cancer-related mortality [1,2]. Ovarian cancer is often asymptomatic in early stages [3,4] and presents nonspecific symptoms in subsequent stages [3]. Thus, early-stage detection, although desirable, is difficult to achieve.

Cancer involves several alterations and interactions, including genetic, epigenetic, and metabolic [5]. Thus, omics and multiomics technologies are a promising source for early-stage diagnosis of cancer, including ovarian cancer, [4]; they could guide prevention, risk-reducing surgeries, and treatments [4]. In addition, genomic data and their use have been growing [6,7]. “Omics” comes from the Latin word *omnis*, which means “everything” [8], and “describes a comprehensive quantitative characterization of a class of molecules in a given biological sample or specimen, aiming to understand the molecular mechanisms and underpinnings underlying the functioning of an organism” [9].

Omics-based studies and the identification of biomarkers provide an important foundation for the development of personalized medicine approaches in cancer and can promote the development of personalized medicine [10,11]. Personalized medicine differs from randomized controlled trials [10]. The latter solve the problem of bias with randomization but provide results based on averages and confidence levels, whereas personalized medicine aims to deal with individual patients’ characteristics [10]. Personalized medicine uses biological information and biomarkers on a molecular level to provide tailored preventive and therapeutic solutions [12]. Moreover, this tailoring approach can also include additional clinical information to improve these solutions [13]. It encompasses not only diagnosis and treatment but also substages such as prognosis, presymptomatic testing, and risk and recurrence assessment [7].

The literature highlights the advantages of a multiomics approach over a mono-omics one in cancer research [14,15]. A multiomics design provides a more comprehensive perspective and insights on the interactions of different types of omics [14]. Specifically, “multiomics approaches in clinical oncology integrate data from various molecular levels to enhance precision medicine” [14]. On this basis, studies on different types of cancer, such as gastric cancer [16,17], pancreatic cancer [18], breast cancer [19-22], pancancer [23-25], and ovarian cancer, have adopted a multiomics perspective.

In this context, the role of AI and machine learning (ML) has been highlighted as they can assess the large volumes of data that a multiomics study entails [14]. A multiomics ML approach provides a better understanding of the phenomena but, at the same time, introduces several challenges. Multiomics studies require integrating imbalanced [26], heterogeneous [4], and high-dimensional datasets [3,27] with limited sample sizes [3,13,27] that could lead to overfitting, limited generalizability, and inaccurate results [15] and capture interactions among genes [28]. In addition, different ML techniques have been useful to predict survival groups in ovarian cancer, with those based on deep learning being the most promissory ones [15,29,30].

On the basis of this, the focus of the current study was on assessing ovarian cancer multiomics data that differentiate survival [3,15] risk groups [31]. To do so, an ensemble of ML techniques was used. Graph neural networks (GNNs) and graph convolutional neural networks (GCNNs) allow for the capture of interactions among different omics [28,32], their biological associations, and patterns of cooperation among features [33]. Data augmentation has been proposed as one strategy to address limited sample sizes in deep learning [13]. In the present study, however, variational autoencoders (VAEs) were used exclusively for nonlinear dimensionality reduction and latent representation learning.

### Literature Review

Predicting survival rates of cancer is an important topic that could allow for the better management of this disease [34] and healing therapies [34]. These predictions could include overall survival, recurrence survival, progression survival, or treatment response [30]. In addition, different types of ML algorithms have been used for this purpose, including models such as random forest, support vector machines, Extreme Gradient Boosting (XGBoost), and deep learning techniques such as GNNs [30] and GCNNs. In addition, the literature acknowledges the Cox proportional hazards (CoxPH) model and its variations as an important and widely used framework [34].

Several studies have aimed to make prognoses or predict survival rates or survival groups in different types of cancer, including ovarian cancer. Among those studies that adopted a multiomics approach, such as the current inquiry, some of them assessed the problem of data [15,20,29,35]. Zhang et al [29] used principal component transformation for dimensionality treatment to improve the performance of the model. In their study, deep learning algorithms obtained better predictive power than decision trees and random forest. Cox regression was used to identify molecular features related to patients’ survival rates.

There are several techniques to deal with the so-called curse of dimensionality in cancer-related studies. Some of them are component or factor based [36], such as principal component analysis [37-39] and principal component transformation [29]. Other methods are projection based, such as isometric mapping, uniform manifold approximation and projection, or t-distributed stochastic neighbor embedding [36]. Modern complex techniques such as autoencoders and VAEs have also been applied for data integration [40] and dimensionality reduction [35,41-43]. The literature points out that there are several variations of VAEs [6]. Similar to autoencoders, a VAE encompasses an encoder and a decoder. The encoder, through multiple layers, produces an embedding vector in the latent space; this latent vector is then decoded, seeking to reconstruct the input data as well as possible [6], reducing their dimensionality. In addition, the latent space of a VAE is regulated based on a known distribution [6].

Studies that predicted survival rates of ovarian cancer using multiomics data have also used autoencoders or VAEs for data integration and dimensionality reduction [15,35]. In the study by Jiang et al [15], a multilayer perceptron was subsequently used to construct a prognostic prediction index for stratifying patients with ovarian and breast cancer into high- and low-risk groups. This model relied on deep learning methods and not on traditional ones such as CoxPH and its variations, random survival forest, XGBoost, and XGBoost with accelerated failure time. Hira et al [35] also propounded a special type of VAE—maximum mean discrepancy VAE—to integrate multiomics data while treating their imbalance, heterogeneity, and high dimensionality. The compressed features of the model were used to cluster samples into molecular subtypes and cancer or noncancer groups. Then, an artificial neural network classified cancer samples and molecular subtypes. The survival analysis included identifying survival subgroups based on univariate CoxPH and clustering samples using the k-means clustering algorithm. The model then predicted survival groups with a support vector machine–based classifier and made a potential prognosis of biomarkers. Jiang et al [15] and Hira et al [35] used the dataset from The Cancer Genome Atlas (TCGA).

The literature has remarked on the relevance of GNNs for biological data assessment. GNNs can model the complexity of data [44], including molecular data such as omics, biological processes, and their interactions [45]. GNNs combine graph structures with the prediction power of deep learning techniques and have different derivations, such as GCNNs [46]. Graph structures are a combination of nodes and edges, where edges provide information about the relationships between nodes [46]. Node classification, edge classification, and link prediction are three main functions that have been related to GNNs [44]. Survival analysis has been one of the uses of GNNs in the topic of cancer [34,46].

In the field of ovarian cancer survival prediction, GNNs have been used with omics data [32] and other types of information, such as laboratory information, vital signs, and treatment records [34]. Wang et al [32] propounded a prognosis model, DFASGCNS (Dual Fusion Channels and Stacked Graph Convolutional Neural Network), for ovarian cancer based on a stacked GCNN and dual-fusion channels. The stacked GCNN allowed the model to better depict the interactions of multiomics data. This study also used the TCGA dataset.

Studies about cancer survival prediction have propounded ensemble models that combine GNNs or GCNNs and VAEs to achieve more robust results [20,47]. Breast cancer was the topic of one of these inquiries [20]. This study used three ensemble models: long short-term memory, VAE, and GCNN. They were optimized using stochastic gradient descent. The VAE compressed the high-dimensional data into a lower-dimensional space. The dataset was taken from the Molecular Taxonomy of Breast Cancer International Consortium. The model obtained 98% accuracy for the optimized long short-term memory model. In addition, Zhang et al [47] sought to improve the accuracy of GNNs while facing the problem of the limited number of neighboring genes in the data. This study propounded a model called LAGProg, which used a conditional VAE as a generative model to augment the features. Survival predictions were made based on GCNN and a Cox proportional risk network. The data were taken from TCGA and included 15 datasets, none of them related to ovarian cancer. It is relevant to mention that this study found 13 prognostic markers related to breast cancer. No similar studies focused on ovarian cancer survival prediction were found. Table 1 summarizes the related studies and highlights the gap that this work aimed to address.

**Table 1.** Related studies in cancer survival prediction using multiomics data.

Studies	Multiomics data use	Dimensionality reduction	Graph-based interactions	ML^a^/DL^b^ model	Ovarian cancer focus
Wang et al [32]	Yes	No	Yes	GCNN^c^	Yes
Jiang et al [15]	Yes	Yes (VAE^d^)	No	VAE	Yes
Zhang et al [29]	Yes	Yes (PCT^e^)	No	ML	Yes
Hira et al [35]	Yes	Yes (MMD-VAE^f^)	No	ANN^g^	Yes
Mahmoud et al [20]	Yes	Yes (VAE)	Yes	GCNN+LSTM^h^	No
Zhang et al [47]	Yes	Yes (VAE)	Yes	GCNN	No
Our study	Yes	Yes (VAE)	Proposed GCNN	VAE	Yes

a ML: machine learning.

b DL: deep learning.

c GCNN: graph convolutional neural network.

d VAE: variational autoencoder.

e PCT: principal component transformation.

f MMD-VAE: maximum mean discrepancy VAE.

g ANN: artificial neural network.

h LSTM: long short-term memory.

### Research Gap

A review of current ovarian cancer and multiomics survival modeling literature revealed that existing studies typically address these methodological challenges in isolation. Specifically, (1) limited sample sizes continue to constrain the development of robust deep learning models for multiomics survival analysis, whereas the potential of generative models for representation learning remains underexplored; (2) high dimensionality and cross-modality heterogeneity are often managed through feature selection or linear dimensionality reduction methods rather than through deep nonlinear representation learning; (3) biological interaction modeling using GCNNs is generally explored in single-omics or pathway-focused settings rather than as part of integrated multiomics survival analysis frameworks; and (4) survival risk group identification from latent multiomics representations is rarely integrated with deep generative representation learning within a unified analytical framework.

To our knowledge, few previous studies on ovarian cancer have proposed an integrated framework that combines VAE-based nonlinear representation learning, graph-based interaction modeling, and survival stratification within a unified analytical pipeline. The present study developed and evaluated a VAE-based framework for multiomics survival stratification and introduced a graph-based extension as a biologically motivated direction for future research.

### Research Objectives

This study aimed to identify prognostically distinct patient subgroups in ovarian cancer through multiomics integration and deep representation learning. To accomplish this, we pursued the following objectives:

- Mitigate the challenges associated with high-dimensional, heterogeneous multiomics data through VAE-based nonlinear representation learning
- Compress 99,322 molecular features into a compact latent representation suitable for downstream survival modeling
- Propose the integration of GCNNs to model relational biological information as a future extension of the framework
- Identify survival-associated patient subgroups through unsupervised k-means clustering of latent representations, followed by CoxPH modeling and Kaplan-Meier (KM) survival analysis to evaluate prognostic relevance

## Methods

### Ethical Considerations

This study is a secondary analysis of publicly available, deidentified data from TCGA accessed via the University of California, Santa Cruz (UCSC), Xena platform [48,49]. No primary data collection, participant recruitment, intervention, or direct contact with human participants was performed, and the researchers did not have access to information permitting individual identification. The Research Ethics and Scientific Integrity Office of the Pontificia Universidad Católica del Perú reviewed the project and determined that it did not require review by any of the university’s research ethics committees because it did not qualify as research involving human participants, animals, or ecosystems (certificate 018-2026-NR/OETIIC/PUCP; August 28, 2026) [50]. TCGA data were accessed and used in accordance with the applicable TCGA human participant protection and data access policies [48].

### Pipeline

**Figure 1.**
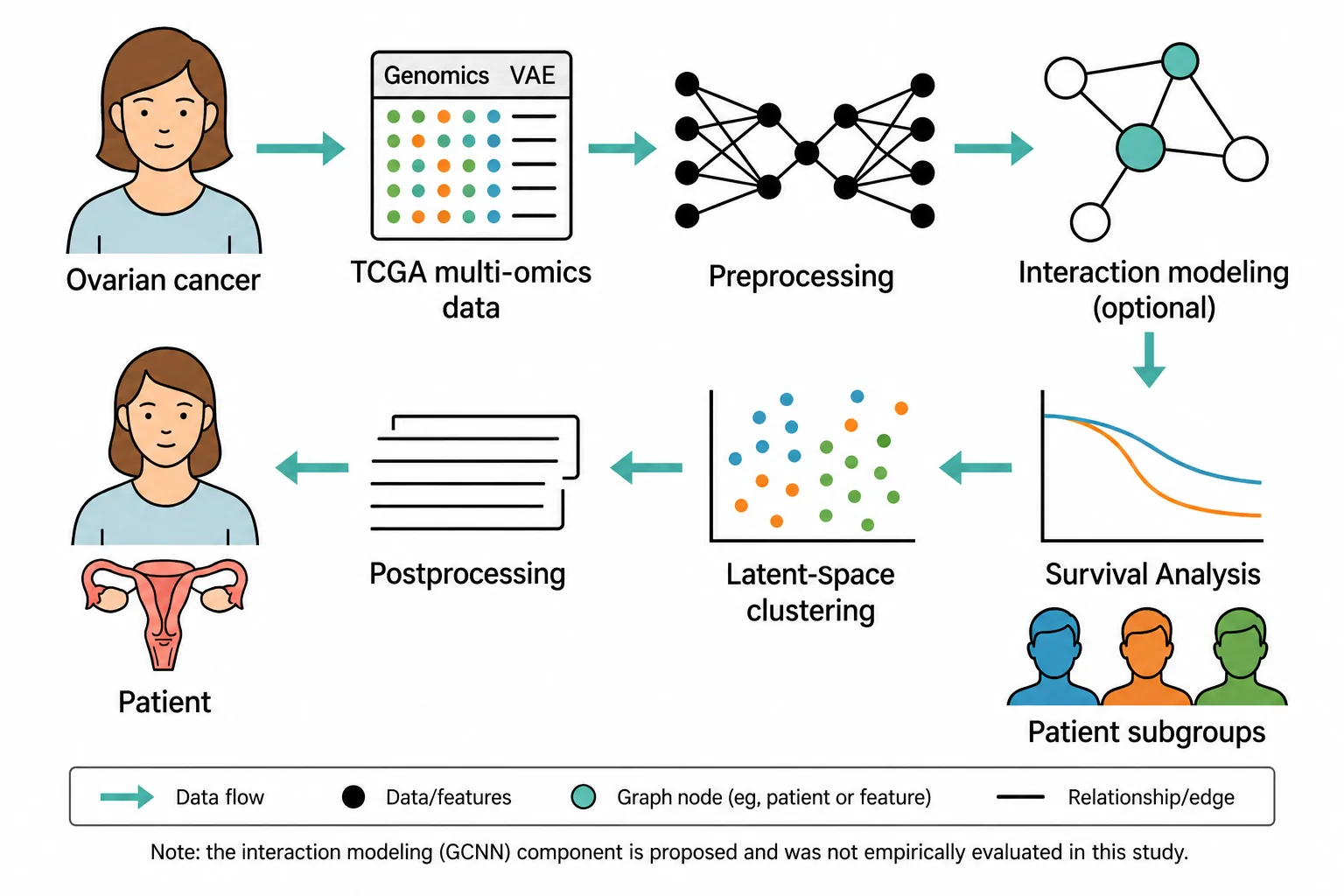
Overview of the proposed multiomics analysis pipeline. GCNN: graph convolutional neural network; TCGA: The Cancer Genome Atlas; VAE: variational autoencoder.

Our analytical pipeline comprises 5 sequential stages, spanning raw multiomics preprocessing, latent representation learning, survival-based patient stratification, and model evaluation (Figure 1). The pipeline includes TCGA multiomics preprocessing; VAE-based nonlinear dimensionality reduction and latent-space representation learning; an optional GCNN component proposed for modeling interaction-aware representations but not empirically evaluated in this study; and downstream survival stratification through k-means clustering, CoxPH modeling, and KM survival analysis. The main components of the 5-stage analytical pipeline are summarized in Textbox 1.

Textbox 1. **Components of the 5-stage pipeline.**

**Stage 1: data acquisition and preprocessing**

- Retrieval of The Cancer Genome Atlas ovarian cancer multiomics data, including genomic, transcriptomic, and epigenomic layers [35]
- Harmonization of patient identifiers and integration of the omics and clinical survival datasets
- Handling of missing values using established imputation strategies [51]
- Detection and treatment of outliers following standard preprocessing practices [51]
- Featurewise normalization to harmonize measurement scales across omics modalities [29]

**Stage 2: latent representation learning via variational autoencoder (VAE)**

- Integration of the multiomics modalities into a unified patient-level feature matrix
- Dimensionality reduction of the combined 99,322 input features into a compact latent representation using a VAE
- Optimization of the VAE using reconstruction loss and Kullback-Leibler divergence regularization
- Extraction of patient-level latent representations for downstream clustering and survival analysis

**Stage 3: proposed graph-based extension**

- Conceptual construction of a graph structure representing patient-level similarity or feature-level biological associations
- Proposed definition of an adjacency matrix to encode structural relationships among graph nodes
- Proposed application of a graph convolutional neural network to refine the VAE-derived latent representations by incorporating graph-structured relational information
- This graph-based component was not implemented or empirically evaluated in the present study; consequently, all reported downstream analyses were conducted exclusively using the VAE-derived latent representations

**Stage 4: survival-based patient stratification**

- Unsupervised k-means clustering of the VAE-derived patient-level latent representations
- Evaluation of candidate clustering solutions using silhouette analysis, with *k*=2 selected as the optimal solution
- Cox proportional hazards (CoxPH) modeling to assess the association between cluster membership and overall survival
- Kaplan-Meier (KM) estimation and log-rank testing to evaluate differences in overall survival between the 2 identified patient subgroups

**Stage 5: model evaluation**

- Silhouette score assessment to quantify cluster compactness and separation; the selected 2-cluster solution achieved a silhouette score of 0.272
- Assessment of VAE reconstruction performance to evaluate the fidelity of the learned latent representation
- Evaluation of the CoxPH hazard ratio, CI, statistical significance, and proportional hazards assumption
- KM survival curve comparison and log-rank testing to evaluate the prognostic relevance of the identified patient subgroups

### Data Sources and Multiomics Integration

We analyzed ovarian cancer multiomics data from TCGA obtained via the UCSC Xena data portal. The dataset included three omics layers: (1) genomics (copy number variation), (2) transcriptomics (RNA sequencing and gene expression arrays), and (3) epigenomics (DNA methylation). We downloaded sample-level matrices, where rows correspond to molecular features and columns correspond to patient samples.

The downstream analyses were conducted on the corrected full matched cohort of 291 patients (n=241, 82.8% events [deaths]; n=50, 17.2% censored). An initial barcode-matching error resulted in an incorrectly reduced cohort of 49 patients: clinical files use participant-level barcodes (12-character prefix), whereas molecular files use aliquot-level barcodes; the merge was incorrectly performed on the full string rather than the prefix, causing most matches to fail silently. After correction, 291 patients were retained. The data processing scripts and materials supporting reproducibility are available in the public GitHub repository [52].

### Sample Attrition and Cohort Harmonization

A complete account of sample attrition is provided to ensure transparency and reproducibility. The Cancer Genome Atlas Ovarian Cancer (TCGA-OV) initial dataset comprised 292 patients with matched multiomics molecular profiles, corresponding to 99,322 molecular features across the selected omics layers. Following cohort harmonization, of the 292 patients, 1 (0.3%) was excluded because a valid participant-level match between the clinical and molecular datasets could not be established. Consequently, the final matched cohort consisted of 291 patients.

Inclusion criteria were as follows: (1) availability of DNA methylation (HumanMethylation450 array), RNA sequencing (Illumina HiSeq), and copy number variation (SNP6 array) data; and (2) availability of overall survival time and event status.

Exclusion criteria were as follows: (1) missing data in any omics layer after quality filtering, (2) overall survival time of 0 or less, and (3) inability to establish a valid participant-level match between the clinical and molecular datasets.

### Preprocessing, Normalization, and Quality Control

Prior to model training, we applied a standard preprocessing pipeline. Features with excessive missingness were removed, and residual missing values were imputed using simple statistics (mean or median) within each omics layer. All features were subsequently standardized to zero mean and unit variance using training set statistics to mitigate scale differences between omics types and to stabilize optimization.

### VAE for Nonlinear Dimensionality Reduction and Representation Learning

To address the high dimensionality and limited sample size of the integrated dataset, we used a VAE as a nonlinear latent representation learning model. Let *x* ∈ *R^D^* denote the concatenated multiomics profile for a patient. The VAE consists of an encoder network $q_{\phi }\left(z|x\right)$ and a decoder network $p_{\theta }\left(x|z\right)$:


(1) $$q_{\phi }\left(z|x\right)=N\left(\mu_{\phi }\left(x\right),diag\left(\sigma_{\phi }^{2}\left(x\right)\right)\right),$$

(2) $$p_{\theta }\left(x|z\right)=N\left(f_{\theta }\left(z\right),\sigma^{2}I\right),$$

In these equations, $z\in R^{d_{z}}$ is a low-dimensional latent variable, and $f_{\theta }\left(\cdot \right)$ denotes the decoder network. The VAE was trained by minimizing the following:


(3) $$L_{VAE}\left(x\right)=\|x-\overset{ˆ}{x}{\|}_{2}^{2}-\beta \;E_{q_{\phi }\left(z|x\right)}\left[\log p\left(z\right)-\log q_{\phi }\left(z|x\right)\right]$$

$$L_{recon}=\|x-\overset{ˆ}{x}{\|}_{2}^{2}$$

$$L_{KL}=E_{q_{\phi }\left(z|x\right)}\left[\log p\left(z\right)-\log q_{\phi }\left(z|x\right)\right]$$

We used the Adam optimizer with a learning rate of 10*^−^*^3^ and mini batches of size 32.

After training, the encoder was used to map each patient to a deterministic latent embedding by computing the posterior mean $\mu_{\phi }\left(x_{i}\right)$ directly without sampling. This pipeline does not use data augmentation. No synthetic patient profiles are generated at any stage. All 291 real TCGA-OV patients are used for VAE training, latent embedding extraction, k-means clustering, CoxPH modeling, and KM estimation. No train-test split is applied before VAE training. The VAE’s reparameterization trick (sampling $z∼N\left(\mu_{\phi },diag\left(\sigma_{\phi }^{2}\right)\right)$) is used only during the forward pass for training; at inference time, the deterministic posterior mean $\mu_{\phi }\left(x_{i}\right)$ is used as each patient’s embedding to ensure reproducible, noise-free representations.

### GCNN

#### Proposed Graph-Based Extension

To explicitly capture molecular interactions and cross-omics relationships, the integrated multiomics data were modeled as a graph. Nodes represented molecular features (eg, genes or methylation probes), whereas edges encoded prior biological relationships, including coexpression, pathway comembership, or correlation thresholds computed from the training data. This process generated an adjacency matrix $A\in R^{N\times N}$, where *N* denotes the number of selected molecular features represented in the graph.

#### Methodological Note

Although a GCNN architecture is described as a biologically motivated extension for modeling molecular interactions and cross-omics relationships, the experiments reported in this paper were performed using the VAE latent representations alone. Consequently, the GCNN formulation should be regarded as a proposed extension of the framework rather than a validated component of the present pipeline. Future work will evaluate its contribution through dedicated ablation studies and comparative analyses on larger cohorts.

In the proposed framework, a GCNN would learn interaction-aware feature representations. Given the initial node feature matrix ***H***^(0)^, derived from either the VAE latent representation or the selected omics features, each GCNN layer would update the node embeddings according to the following equation:


(4) $$H^{\left(l+1\right)}=\sigma \left({\tilde{D}}^{-\frac{1}{2}}\tilde{A}{\tilde{D}}^{-\frac{1}{2}}H^{\left(l\right)}W^{\left(l\right)}\right),$$

where


$$\tilde{A}=A+I$$

is the adjacency matrix with self-loops,


$${\tilde{D}}_{ii}=\sum_{j}{\tilde{A}}_{ij}$$

is the corresponding degree matrix, ***W***^(*l*)^ is the trainable weight matrix for the *l*th graph convolution layer, and *σ*(·) denotes a nonlinear activation function (rectified linear unit in this study). By stacking multiple GCNN layers, information would be propagated across neighboring nodes, enabling the model to capture both local and higher-order molecular interactions while integrating complementary information across multiple omics modalities.

### Survival Modeling and Risk Grouping

Clinical survival data, including overall survival time and event status, were linked to each patient. CoxPH models were used to assess the association between the learned latent representations and survival outcomes. To derive clinically interpretable survival subgroups, k-means clustering was applied to the latent space, with the optimal number of clusters selected based on silhouette analysis. Patients were subsequently assigned to one of the identified latent clusters. KM survival curves were estimated for each cluster, and the log-rank test was used to evaluate the statistical significance of differences in overall survival between the resulting patient groups.

### Implementation Details

All analyses were implemented using Python (Python Software Foundation) in a reproducible Google Colab environment. Data handling and preprocessing used pandas and scikit-learn (Google Summer of Code project); the VAE model was implemented in PyTorch (Meta AI). Clustering and silhouette scores were obtained using scikit-learn, whereas CoxPH and KM analyses were conducted using standard survival analysis libraries. The complete code and configuration files are available at the UCSC Xena platform [52].

## Results

### Latent Representation of Multiomics Data

**Figure 2.**
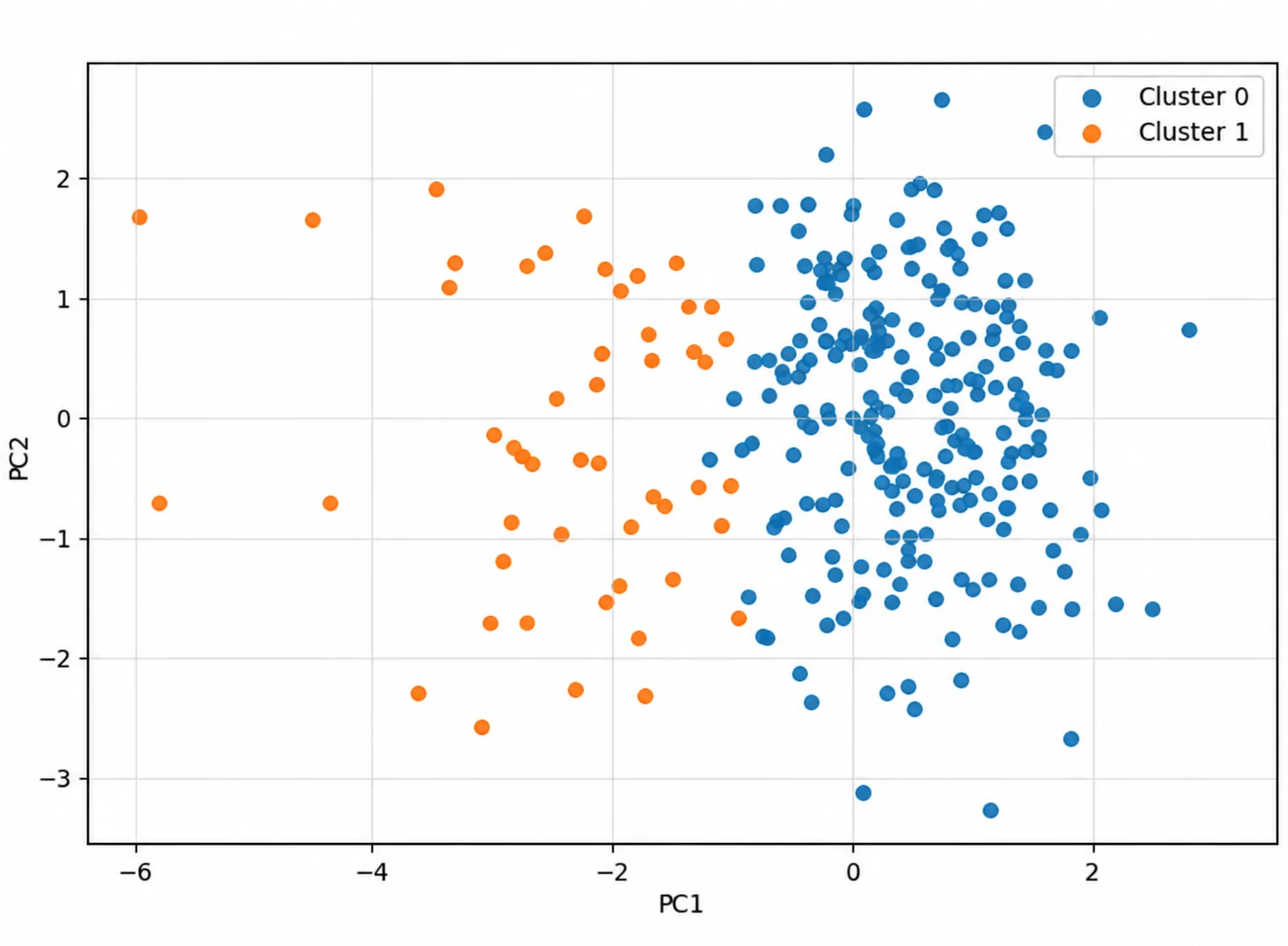
Principal component analysis projection of the variational autoencoder–derived latent representations showing the 2 patient clusters identified through k-means clustering (*k*=2; silhouette score=0.272). PC1: principal component 1; PC2: principal component 2.

The refined VAE was trained using the final matched cohort of 291 TCGA-OV patients over 50 training epochs. The model learned an 8D latent representation for each patient, providing a compact embedding of the integrated multiomics data. Prior to clustering, the latent features were standardized to zero mean and unit variance to ensure equal contribution of each latent dimension to the clustering process. Figure 2 shows a 2D principal component analysis projection of the resulting latent space.

### Survival Modeling With CoxPH

**Figure 3.**
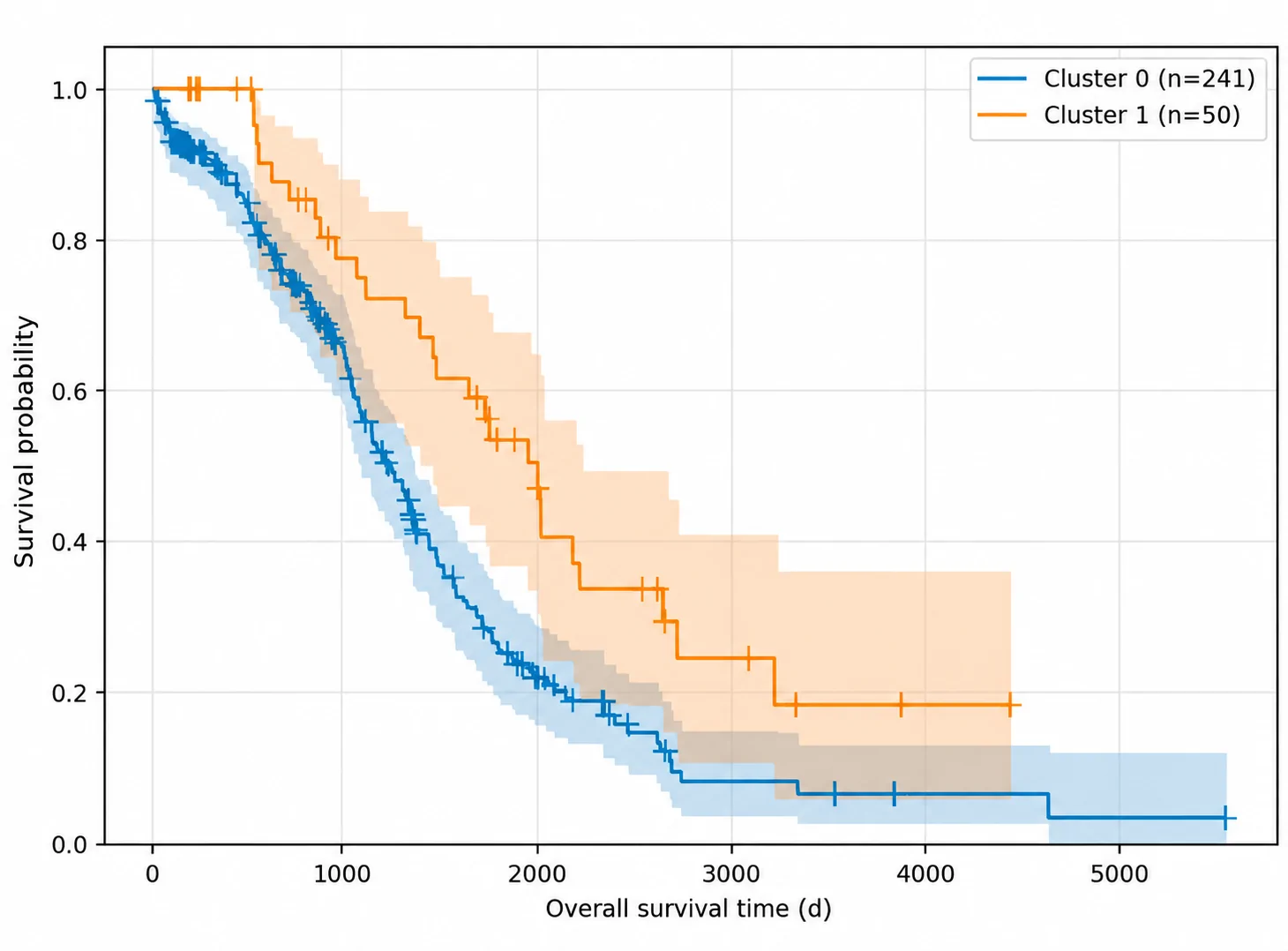
Kaplan-Meier survival curves stratified by the 2 latent clusters identified from the variational autoencoder latent representations. Cluster 1 (n=50) exhibited significantly improved overall survival compared with cluster 0 (n=241). Shaded regions represent the 95% CIs, and check marks denote censored observations. The difference between survival curves was statistically significant according to the log-rank test (*P*=.002).

To quantify the prognostic significance of the latent clusters, a CoxPH model was fitted using cluster assignment as the predictor and overall survival as the outcome. The estimated hazard ratio (HR) was 0.519 (95% CI 0.343‐0.785; *P*=.002), indicating that patients assigned to cluster 1 exhibited an approximately 48% lower hazard of death than those in cluster 0. This finding demonstrates a statistically significant association between the latent cluster assignment and overall survival. The proportional hazards assumption was assessed using Schoenfeld residuals. The global test was not statistically significant (*P*≈.28), and no systematic temporal trends were observed, supporting the validity of the CoxPH model (Figure 3).

### Unsupervised Clustering and Survival Stratification

The optimal number of clusters was determined by evaluating candidate solutions with *k*=2 to *k*=8 using silhouette analysis. The resulting silhouette scores were 0.272, 0.110, 0.140, 0.154, 0.162, 0.156, and 0.146, respectively. The highest silhouette score (0.272) was obtained for *k*=2, which was therefore selected as the optimal clustering solution. The resulting k-means clustering partitioned the cohort into cluster 0 (n=241) and cluster 1 (n=50). Figure 3 shows the KM survival curves for the 2 latent clusters. Patients assigned to cluster 1 exhibited significantly better overall survival than those in cluster 0 (*P*=.002). The log-rank test demonstrated a statistically significant difference between the survival distributions (*χ*^2^=10.0; *P*=.002), confirming that the VAE-derived latent clusters capture prognostically distinct patient groups.

## Discussion

### Principal Results

We analyzed three major molecular layers—genomics, transcriptomics, and epigenomics—that together capture both stable and dynamic aspects of tumor biology. After preprocessing and harmonization, the multiomics matrices were integrated into a unified representation using a VAE, which compressed the high-dimensional feature space into a lower-dimensional latent manifold while preserving major sources of biological variation.

The pipeline also proposes an optional GCNN component to incorporate relational structure among molecular features. The rationale for the VAE and GCNN combination lies in complementary limitations: the VAE treats molecular features as conditionally independent given the latent code, discarding relational structure such as gene coexpression patterns, whereas the GCNN explicitly propagates information along edges of the molecular graph. However, this theoretical complementarity was not empirically validated in the present study. The GCNN is best understood as a theoretically motivated proposed extension whose empirical utility remains to be demonstrated.

The revised analyses demonstrate that the VAE pipeline applied to the full cohort of 291 patients identified two statistically distinct patient subgroups with significantly different overall survival (log-rank *P*=.002; HR 0.519, 95% CI 0.343‐0.785; Cox model *P*=.002). Silhouette-guided cluster selection identified *k*=2 as the optimal solution (silhouette score=0.272). The proportional hazards assumption was verified using Schoenfeld residuals (global test *P*≈.28), supporting the validity of the CoxPH model. These findings demonstrate that the VAE-derived latent representations capture prognostically distinct patient groups within the TCGA ovarian cancer cohort.

Taken together, these results demonstrate that the proposed VAE pipeline identifies statistically significant prognostic subgroups when applied to the full TCGA-OV cohort of 291 patients. The combination of silhouette-guided cluster selection, KM survival analysis, and CoxPH modeling consistently supports the prognostic relevance of the VAE-derived latent representations. These findings provide evidence that unsupervised representation learning can identify clinically meaningful patient subgroups from integrated multiomics data and establish a foundation for future extensions incorporating graph-based learning and biological pathway analysis.

### Comparison With Prior Work

Our findings are consistent with those of previous studies demonstrating the utility of deep learning and VAEs for modeling the complex structure of multiomics data in cancer prognosis. Jiang et al [15] also investigated ovarian cancer using TCGA-derived multiomics data and reported that a VAE-based framework effectively captured complex molecular patterns through latent feature learning and data augmentation. Although the methodological approaches differ, the study by Jiang et al [15] and the present study support the value of representation learning for integrating heterogeneous multiomics data and improving prognostic stratification in ovarian cancer. The present study further extends this evidence by demonstrating that VAE-derived latent representations identify statistically significant prognostic subgroups within the full matched TCGA-OV cohort of 291 patients.

### Limitations

First, the patient matching procedure between the clinical and molecular datasets was revised to ensure correct harmonization of TCGA participant identifiers. Consequently, all analyses in this revised manuscript were performed using the full matched TCGA-OV cohort of 291 patients (n=241, 82.8% events and n=50, 17.2% censored observations), replacing the previously analyzed subset of 49 patients. This correction substantially increases statistical power and strengthens the reliability of the reported findings.

Second, no formal ablation study was conducted to quantify the individual contribution of each component of the proposed pipeline. Specifically, the survival stratification performance was not compared across (1) raw multiomics features, (2) VAE-derived latent embeddings, and (3) VAE embeddings augmented with graph-based learning. Consequently, the incremental benefit of each component, particularly the proposed GCNN extension, could not be independently quantified.

Third, the VAE was trained in an unsupervised manner without incorporating clinical outcome information during representation learning. Future work may investigate supervised or semisupervised approaches that integrate survival objectives into the latent-space optimization or incorporate molecular interaction networks directly within the representation learning process.

### Future Work

Future research should extend the present framework in several directions. First, external validation using independent multiomics ovarian cancer cohorts will be essential to assess the generalizability and clinical applicability of the proposed methodology. Second, formal ablation studies should be conducted to quantify the contribution of each component of the analysis pipeline, including comparisons among raw multiomics features, VAE-derived latent representations, and future graph-based extensions. Third, supervised or semisupervised representation learning approaches, such as survival-informed VAEs, may further improve prognostic stratification by incorporating clinical outcome information during latent-space optimization. Finally, future work may integrate GNN architectures and biologically informed gene-gene or patient-patient similarity networks to better capture molecular interactions and enhance both predictive performance and model interpretability.

### Conclusions

This study demonstrates that a VAE-based framework can integrate multiomics data to identify prognostically distinct patient subgroups in ovarian cancer. Applied to a matched TCGA-OV cohort of 291 patients, the proposed pipeline identified two latent clusters with significantly different overall survival, as demonstrated through KM analysis (log-rank *P*=.002) and CoxPH modeling (HR 0.519, 95% CI 0.343‐0.785; *P*=.002). The optimal two-cluster solution was supported by silhouette analysis, and the proportional hazards assumption was satisfied, supporting the validity of the CoxPH model. These findings demonstrate the potential of unsupervised deep representation learning for survival stratification using integrated multiomics data. Future work should focus on external validation in independent cohorts, comprehensive biological characterization of the identified latent clusters, formal ablation studies to quantify the contribution of individual pipeline components, and the evaluation of graph-based learning approaches to further enhance predictive performance and interpretability.

## Abbreviations

CoxPH: Cox proportional hazards
DFASGCNS: Dual Fusion Channels and Stacked Graph Convolutional Neural Network
GCNN: graph convolutional neural network
GNN: graph neural network
HR: hazard ratio
KM: Kaplan-Meier
ML: machine learning
TCGA: The Cancer Genome Atlas
TCGA-OV: The Cancer Genome Atlas Ovarian Cancer
UCSC: University of California, Santa Cruz
VAE: variational autoencoder
XGBoost: Extreme Gradient Boosting
